# Exploitation of Outgoing and Incoming Telephone Calls in the Context of Circadian Rhythms of Social Activity Among Elderly People: Observational Descriptive Study

**DOI:** 10.2196/13535

**Published:** 2020-11-26

**Authors:** Timothée Aubourg, Jacques Demongeot, Hervé Provost, Nicolas Vuillerme

**Affiliations:** 1 Orange Labs Meylan France; 2 Univ Grenoble Alpes AGEIS Grenoble France; 3 LabCom Telecom4Health Univ Grenoble Alpes & Orange Labs Grenoble France; 4 Institut Universitaire de France Paris France

**Keywords:** circadian rhythms, phone call detail records, older population, digital phenotype

## Abstract

**Background:**

In the elderly population, analysis of the circadian rhythms of social activity may help in supervising homebound disabled and chronically ill populations. Circadian rhythms are monitored over time to determine, for example, the stability of the organization of daily social activity rhythms and the occurrence of particular desynchronizations in the way older adults act and react socially during the day. Recently, analysis of telephone call detail records has led to the possibility of determining circadian rhythms of social activity in an objective unobtrusive way for young patients from their outgoing telephone calls. At this stage, however, the analysis of incoming call rhythms and the comparison of their organization with respect to outgoing calls remains to be performed in underinvestigated populations (in particular, older populations).

**Objective:**

This study investigated the persistence and synchronization of circadian rhythms in telephone communication by older adults.

**Methods:**

The study used a longitudinal 12-month data set combining call detail records and questionnaire data from 26 volunteers aged 70 years or more to determine the existence of persistent and synchronized circadian rhythms in their telephone communications. The study worked with the following four specific telecommunication parameters: (1) recipient of the telephone call (alter), (2) time at which the call began, (3) duration of the call, and (4) direction of the call. We focused on the following two issues: (1) the existence of persistent circadian rhythms of outgoing and incoming telephone calls in the older population and (2) synchronization with circadian rhythms in the way the older population places and responds to telephone calls.

**Results:**

The results showed that older adults have their own specific circadian rhythms for placing telephone calls and receiving telephone calls. These rhythms are partly structured by the way in which older adults allocate their communication time over the day. In addition, despite minor differences between circadian rhythms for outgoing and incoming calls, our analysis suggests the two rhythms could be synchronized.

**Conclusions:**

These results suggest the existence of potential persistent and synchronized circadian rhythms in the outgoing and incoming telephone activities of older adults.

## Introduction

### Background

The lives of humans are deeply structured on a daily basis, which both reflects and contributes to the emergence of regular rhythms of activity, the so-called *circadian* (ie, 24 hours) rhythms [1]. Circadian rhythms help individuals affirm and maintain stability in their lives. Behind the apparent regularity, circadian rhythms reflect complex processes with multiple origins. On one hand, at the endogenous level, they stem directly from a biological clock, known as the suprachiasmatic nucleus [2]. Depending on the individual, this internal clock has an almost 24-hour period and regulates the functioning of the entire body throughout the day. On the other hand, at the exogenous level, these biological time posts are entangled with physical and social cues [3], such as the day-night alternation [4] and the daily social interactions [5] that occur between individuals and their social network. These external “time givers,” known under the German word “Zeitgeberen,” permit individuals to adapt themselves to their physical and social environments by aligning their daily activities to a precise and persistent 24-hour clock [4]. Overall, this close synchronization results from the continuous interplay between the fundamental biological and social time cycles in the lives of all individuals [6].

According to the social zeitgeber theory [7], a change in social cues or the occurrence of particular social events, such as life stress [7] and affective episodes [8], may have severe repercussions on these biological and social times. Notably, social changes have the potential to weaken the stability of individuals’ daily lives [7], and the resulting instability may be associated with the social jet-lag phenomenon, in which the biological and social circadian rhythms are desynchronized [9]. In the field of health monitoring, such desynchronization provides important information about an individual’s health status because it may be directly associated with health complications, such as sleep disruption [10], depression [11], and cardiometabolic problems [12]. For health professionals, it is important to properly manage these social disruptions. Thus, their detection may permit us to better understand the mechanisms underlying these severe health issues and thereby prevent them.

For seniors in particular, monitoring circadian rhythms is of importance, as amply demonstrated by the rich literature on the subject, in which these rhythms were shown to change greatly with age (eg, [13-16]). Such changes are generally due to a degradation in temporal organization in the life of seniors caused by age-related alterations that affect their biological clock [15]. These alterations are reflected, for instance, by the chronotype shift that usually appears with aging [14]. This behavioral drift can be considered as a time-induced transition in a senior’s preferences for being active during the day, generally in the morning [17]. At the social level of seniors, this chronotype shift may notably be associated with alterations in their social routines, such as those imposed by social-rhythm stereotyping [18]. Moreover, the chronotype shift may be accompanied by the nonreception of social cues caused by the aging process, leading to a decline in social interactions [19,20], inconsistent meal times [21,22], or fragmented sleep [17]. Finally, these social disruptions may cause feedback and impact the biological state of the individual [7]. Such feedback may notably maintain or worsen the dysregulation of biological circadian rhythms, thus facilitating the health issues mentioned above, which may dramatically impact an older person’s life, as evinced by Alzheimer disease [23-25], Parkinson disease [26], or dementia [21]. Thus, it is of importance to investigate the social aspects of circadian rhythms and their disruption in the elderly population to better understand how these rhythms, or lack thereof, affect the biological clock of seniors and the mechanisms that trigger health problems.

### Prior Studies

A body of literature is presently emerging around mobile health sensing, including the effect of circadian rhythms and social behaviors (eg, [27-29]). Much of this literature discusses the potential of using telephones to enhance the traditional health care system by exploiting health-related data generated by telephone use [30,31]. In particular, analyses of telecommunication data, such as telephone call detail records, enable the detection of circadian rhythms of social activity [32-36]. For example, the recent work of Aledavood et al [32] demonstrated the existence of a circadian temporal signature of outgoing telephone call activity in a young population. Additionally, with the same population sample, Aledavood et al [33] showed that when using a descriptive approach to compare outgoing telephone calls with outgoing text messages, young adults establish persistent temporal signatures whose patterns may differ between voice calls and text messages. In the field of health care research, these fundamental results about digital rhythms suggest that telephones may be used to monitor daily social activity. In recent publications, this sort of exploitation of telephones has provided promising results for monitoring the health status and biological timeframe of individuals [37-40].

Note, however, that it remains unclear whether similar results apply to underinvestigated populations and how they can be implemented. In particular, no studies have heretofore investigated whether the telephone activity of seniors follows a consistent and synchronized circadian rhythm.

### Study Goals

This work was thus specifically designed to determine whether persistent and synchronized circadian rhythms exist in the telephone activity of older adults. To this end, we used a longitudinal data set of 12 successive months combining call detail records and questionnaire data from 21 volunteers over 70 years of age. In this study, we extracted the following four specific telecommunication parameters from the call detail records: (1) recipient of the telephone call (alter), (2) time at which the telephone call began, (3) telephone call duration, and (4) telephone call direction (outgoing or incoming).

## Methods

### Data Collection and Volunteer Recruitment

This study involved a secondary analysis of previously published and unpublished data set analyses [41-43]. Our data set included 12 months of outgoing call detail records for 26 volunteers (20 women and 6 men; median age 84 years, range 71-91 years). Call detail records provided by the local communication service provider were collected from the personal telephones of the volunteers. Each call detail record contained the date, hour, source ID, recipient ID, direction, and duration of the call (in seconds). In addition, volunteers with several telephones registered with their communication service provider (eg, one or more landlines and/or one or more mobile telephones) provided call detail records for all their telephones. Note that the telephone owners and the telephone contacts remained anonymous. Additionally, each volunteer filled in a questionnaire about the people in their telephone social network and classified each of their telephone contacts into one of the following five social categories: family, friend, acquaintance, health professional, and other.

This study and its corresponding experimental protocols were declared to the French Data Protection Authority (CNIL registered data protection officer, France Telecom 2011 n°44). All experimental methods were carried out in accordance with the relevant regulations, and written informed consent was obtained from all participants before data were collected and anonymized.

### Data Preprocessing

Participants did not all enroll in the survey at the same time, so the dates of inclusion varied. Thus, the call detail record data set was filtered to select the time interval when the greatest number of volunteers were actively participating in the study. The call detail record data set was then preprocessed by applying the method described by Saramäki et al [44], which involved selecting only those participants who used their telephones throughout the 12-month observation period, providing a set of 21 individuals (Table 1). Additionally, note that only the incoming telephone calls answered by the volunteer were used in the analysis (ie, incoming telephone calls that went unanswered were removed from the call detail record data set).

**Table 1:** Structure of the data set of call detail records before and after preprocessing.

Variable		Before preprocessing	After preprocessing
**Number of participants**		26	21
	Female	20	16
	Male	6	5
Age (years), range		71-91	71-91
Age (years), mean (SD)		84 (4)	83 (4)
**Total number of calls**			
	Outgoing	19,198	18,338
	Incoming	20,062	17,879
**Average number of calls per individual, first quartile**			
	Outgoing	285	481
	Incoming	579	605
**Average number of calls per individual, median**			
	Outgoing	590	710
	Incoming	718	800
**Average number of calls per individual, third quartile**			
	Outgoing	944	1096
	Incoming	947	992

### Data Analysis

#### Measuring the Daily Rhythms of Telephone Activity

We followed the descriptive approach proposed by Aledavood et al [32] that treats outgoing and incoming telephone calls separately. This approach involves calculating the daily pattern of telephone activity for the study’s entire 12-month data set, which is done by applying a two-step process consisting of (1) coarse graining the time dimension into unique days divided into 24 one-hour time slots and (2) calculating the average fraction of daily calls. The fraction of daily telephone calls for each time slot is given by the following formula:

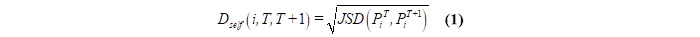



where *n(t)* is the number of calls in time slot *t*.

We followed this approach at the aggregate level to obtain a concise overview of the data set’s structure and trends. Thereafter, to avoid an ecological fallacy [32], the daily rhythms of telephone activity for each individual (ie, ego) was estimated by zooming in to the individual level, as done by Aledavood et al [32].

#### Determining the Consistence of Daily Rhythms of Telephone Activity in Older Adults

To determine the consistence of daily rhythms of telephone calls for each ego, we followed an analytical approach based on the notion of persistence applied separately to outgoing and incoming telephone calls. This notion was proposed by Saramäki et al [44] and applied previously [32-34] to assess the repeatability in time of daily rhythms of telephone activity in accordance with circadian rhythm framing. The approach consists of comparing the stability of estimated daily rhythms at distinct successive time points by applying the below three steps.

(1) Step 1: *Time discretization.* We applied a coarse-graining process [32]. Since the call detail record data set contained 12 months of observations, it was split into three successive 4-month periods called T1, T2, and T3. Note that, although the literature offers no clear consensus regarding the time-interval size, aggregating data into intervals of several months enhances the robustness of the persistence analysis and limits the short-term variations in the patterns of telephone calls [45].

(2) Step 2: *Calculation of daily rhythm*. We calculated the daily rhythm of telephone calls for each ego and for each time interval (T1-T3) by using the same two-step computation described in the previous section.

(3) Step 3: *Persistence of daily rhythm.* The persistence of daily rhythm was determined for each ego by measuring the repeatability of the daily rhythms of telephone call activity over time via a persistence analysis. This analysis involves calculating the stability of each ego’s call patterns in each time period (T1-T3) by using the square root of the Jensen-Shannon divergence dissimilarity measure [35] (see Statistical Tools in the Methods section).

We denote by *D*_self_ the dissimilarity measure of the individual’s daily rhythms between two successive periods. *D*_self_ is given by the following formula:

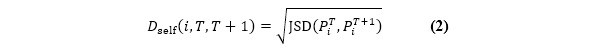



where *P_i_^T^* (*P_i_^T^*^+1^) is the discrete probability distribution of the call fraction of ego *i* at time period *T* (*T+*1) and JSD is Jensen-Shannon divergence.

We denote by 〈*D*_self_〉 the average of *D*_self_ as follows:

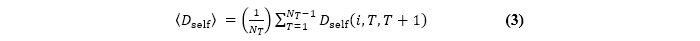



where *N_T_*=3 is the number of time periods.

Thereafter, a reference scale for further comparison was implemented for each ego by calculating, for each time interval (T1-T3), the square root of the Jensen-Shannon divergence between the daily pattern of the given ego and that of every other ego.

We denote by *D*_ref_ the dissimilarity measure between two daily rhythms for two distinct individuals in the same time period. *D*_ref_ is given by the following formula:

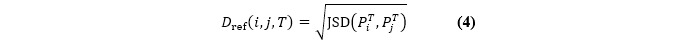



where *P_i_^T^* (*P_j_^T^*) is the discrete probability distribution of call fractions for ego *i* (*j*) at time period *T*, with *i* ≠ *j*, and JSD is Jensen-Shannon divergence.

We denote by 〈*D*_ref_〉 the average of *D*_ref_, which is given as follows:

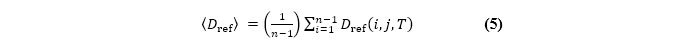



where n=21 is the number of individuals.

Finally, the persistence of daily rhythm for a given ego was evaluated by comparing the average call pattern over time with the average reference scale. Formally, an ego’s daily rhythm is persistent if and only if 〈*D*_self_〉/〈*D*_ref_〉 < 1.

#### Comparing Daily Rhythms of Outgoing and Incoming Telephone Activity

The daily rhythms of outgoing and incoming telephone calls are compared by using the following two steps:

(1) Step 1: *Synchronization assessment*. For each individual and for each time interval (T1-T3), we applied the Kolmogorov-Smirnov comparison test to compare the distribution of outgoing telephone calls during the day with the distribution of incoming telephone calls [46]. The two distributions are considered synchronized if and only if they follow the same law with a *P* value >.05 under the following null hypothesis: “H_0_: the distributions of outgoing and incoming telephone calls follow the same law.”

(2) Step 2: We completed the synchronization assessment by applying a descriptive approach that investigates the alter specificity of the daily rhythms of outgoing and incoming telephone calls to determine whether specific time intervals exist for communicating with specific alters during the day. To this end, we used both the call detail records and questionnaire data for the egos, as performed by Aledavood et al [32]. Based on these pieces of information, the following three questions were addressed: (1) Were there specific hours for communicating with specific alters? (2) What was the fraction of telephone calls to/from the top two alters and who were the top two alters (ie, the alters most frequently in telephone contact with the ego)? (3) What was the variation in the duration of telephone calls between egos and their social network?

In particular, to answer question 1, we analyzed how each ego corresponds with his or her alters through the day by applying the following steps (step 1 and step 2):

(1) Step 1: *Time discretization*. For each ego in each time period (T1-T3), we coarse grained the time dimension into four bins of equal time as follows: night (12 AM-6 AM), morning (6 AM-12 PM), afternoon (12 PM-6 PM), and evening (6 PM-12 AM).

(2) Step 2: *Alter-specificity assessment*. For each ego and for time periods T1 to T3, the alter specificity of the daily rhythms was assessed by comparing the alter structure with that of a null model simulating total randomness in alter specificity. To this end, we estimated the diversity of alters for each 6-hour bin and for each ego by calculating the associated relative entropies (see Statistical Tools in the Methods section) for time periods T1 to T3. This calculation was performed in two steps as follows: (1) Step 2a: *Calculation of origin entropy*. We calculated the origin entropy *H*_orig_ for each ego and for each 6-hour time bin for time periods T1 to T3; (2) Step 2b: *Calculation of relative entropy*. Thereafter, we obtained the relative entropy *H*_rel_ by normalizing *H*_orig_ by the average 〈*H*_ref_〉 of the reference entropy resulting from a null model. This null model is based on the hypothesis that no specific times are associated with telephone calls to specific alters. Thus, a low relative entropy, tending to zero, indicates a relevant alter specificity in the call pattern.

### Statistical Tools

This section presents the following three technical tools used in this study: (1) normalization formulas, (2) Jenson-Shannon divergence, and (3) relative entropy.

#### Normalization

To visualize the peaks and troughs of each subject’s telephone call activity on a single graph, we normalized the daily fraction of their calls to the range (0, 1) by using a unity-based normalization formula [47]. Formally, we let n=21 be the number of participants included in this study and let *p_i_* be a length *t* vector of the daily fraction of calls for subject *i*, with *t* in {0, 1, …, 23} and *i* in {1,2, …, *n*}. We denote by *p_i_*(*t*) the ratio of calls of subject *i* at hour *t*. Then, for each element *p_i_*(*t*) in the vector *p_i_*, we denote by *p_i_*_,normalized_(*t*) its normalized value as follows:

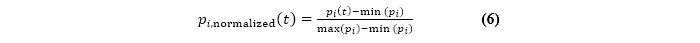



#### Jensen-Shannon Divergence

Jensen-Shannon divergence is a measure of dissimilarity that compares two probability distributions; it is a symmetrical finite-valued version of the Kullback-Leibler divergence. Its square root can be used as a metric for measuring the distance between two probability distributions. Formally, in a discrete context, we have the following:

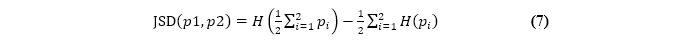



where *p*1 and *p*2 are two discrete probability distributions, *H*(∙) is the Shannon entropy, and JSD is Jensen-Shannon divergence.

#### Entropy Measures

At the individual level, to compare the daily rhythms of outgoing and incoming telephone calls by investigating their alter specificity, we measured the diversity of alters at different hours of the day by calculating the relative entropy. As detailed below, the relative entropy is computed separately for outgoing and incoming telephone calls.

For a given ego *i*, with *i* in {1, 2, …, *n*} and n=21 being the number of participants, we calculated the relative entropy of alter specificity by normalizing the origin entropy by the reference entropy for each 6-hour time interval. This calculation was performed in the following three steps:

(1) Step 1: *Origin entropy.* Formally, let *A_i_* = {a_i1_, a_i2_, …, a_im_} be the set of alters of ego *i*, where *m* gives the size of the social network of ego *i*. For a 6-hour time interval *t*, the origin entropy is as follows:

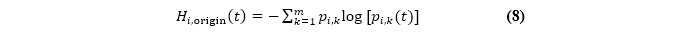



where *p_i,k_*(*t*) is the fraction of calls between ego *i* and the alter *a_ik_* at time interval *t*.

(2) Step 2: *Reference entropy.* We now defined a null model that simulates a system with no specific alter structure. To this end, the number of calls between ego *i* and each alter is preserved, but to simulate randomness, we shuffled the time of the calls in each 2-week period [32]. Thereafter, for each 6-hour interval *t* for this shuffled data set, we calculated the corresponding origin entropy *H_i,origin_*(*t*) as outlined in the previous step. By iterating this process N=1000 times, we obtained the reference entropy [32].

(3) Step 3: *Relative entropy.* To estimate the alter specificity of our original system, we normalized the origin entropy of ego *i* in each time interval *t* by the reference entropy as follows:

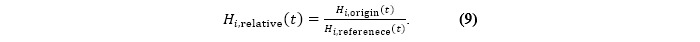



## Results

### Telephone Activity of Older Adults Exhibited Daily Rhythms

**Figure 1:**
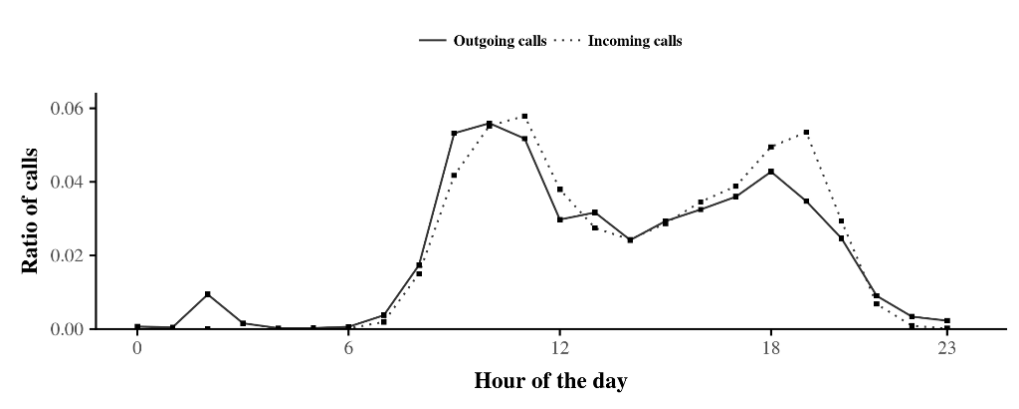
Daily rhythm of telephone calls of older adults at the aggregate level for outgoing and incoming telephone calls. The shape of the curves suggests that similarities exist in how older adults place and receive telephone calls during the day. It also suggests differences, especially at night around 2 AM and in the evening when the fraction of incoming telephone calls is prominent.

Figure 1 shows separately, at the aggregate level, the overall daily rhythms of outgoing and incoming telephone calls for the older adults participating in the study. The results showed that both of these daily rhythms were marked by the following two distinct peaks in activity: (1) a peak at mid-morning around 10 AM after typical waking times and (2) a peak at the end of the day around 6 PM. Both peaks are in accordance with the notion of circadian acrophase in humans, which is usually diurnal [2]. Note that these two peaks are separated by a period of low activity in the afternoon, beginning around 2 PM and matching the typical nap time for elderly people [48]. Additionally, telephone call activity was the slowest during the habitual sleep times in the nocturnal period, in accordance with the circadian nadir phenomena usually occurring at this time [2]. For outgoing telephone calls only, we also noted a small peak in nocturnal activity around 2 AM, which seems rather unusual given the prominent diurnal activity pattern of the data set. For incoming telephone calls, the evening activity peak was noticeably higher than that for outgoing telephone calls. Taken together, these results suggest that the daily rhythm of telephone calls in older adults corresponds with circadian rhythm framing. Interestingly, the daily rhythms for outgoing and incoming telephone calls resembled each other, although they also had their own specific characteristics.

**Figure 2:**
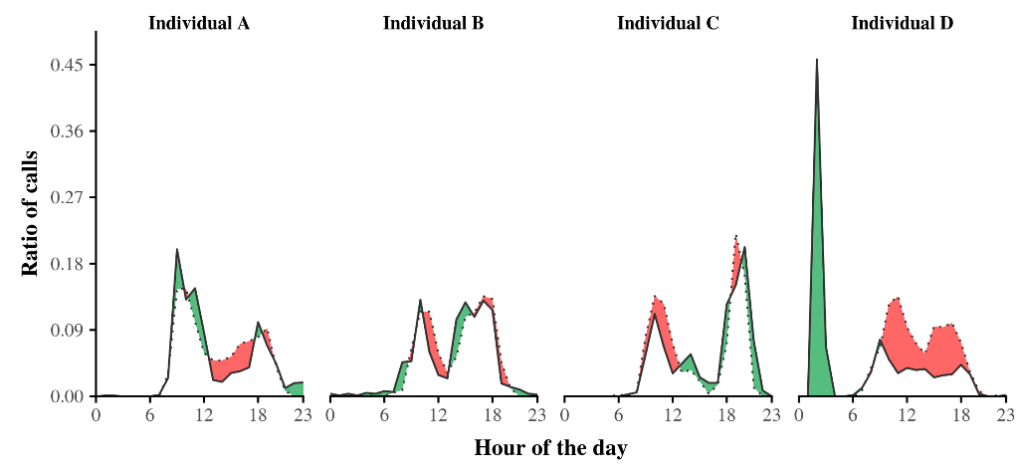
Representative daily rhythms of outgoing and incoming telephone activity for four egos. The solid (dotted) curve shows the outgoing (incoming) call pattern. When the fraction of outgoing telephone calls is greater than the fraction of incoming telephone calls, the difference is shown in green. In the opposite case, the difference is shown in red. The results suggest that both similarities and differences exist in the way the egos place telephone calls and respond to telephone calls during the day. The importance of these observations also appears to vary between egos. In particular, ego D has a prominent nocturnal outgoing telephone call activity that does not appear in the incoming telephone call activity.

To avoid an ecological fallacy [32], we also observed separately the daily telephone activity of each ego. Figure 2 shows the call fractions for four egos, for which the highest peak in telephone activity was in the morning, afternoon, evening, and night. The results suggest not only (1) the existence of daily rhythms for each ego for both outgoing and incoming telephone calls, but also (2) similarities and differences in the way the egos place and receive telephone calls during the day.

**Figure 3:**
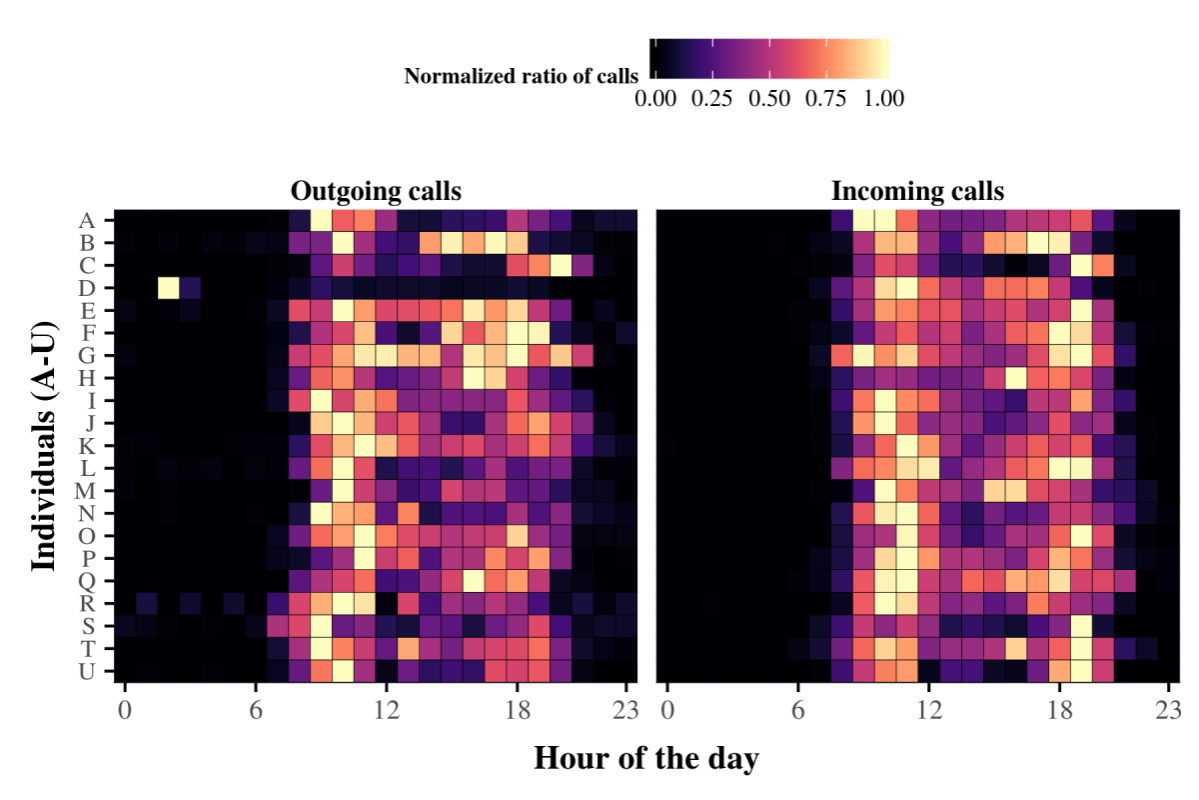
Normalized daily rhythms of outgoing and incoming telephone activity at the individual level. The normalized daily rhythms for each ego are aggregated into a heat map for the outgoing telephone calls (right panel) and for the incoming telephone calls (left panel). The colored squares in each row give the daily rhythm for the telephone calls of one ego with 1-hour resolution (see color scale at the top). Higher fractions of telephone calls correspond to brighter squares. The results show that, in general, most telephone activity occurs during the day rather than at night, except for ego D. Similarities and differences also appear in the way egos place telephone calls versus how they receive telephone calls during the day. Taken together, these observations highlight the potential of telephone activity to reveal the daily rhythms of social activity in older adults.

To put these illustrative results into context, Figure 3 shows the normalized circadian patterns of the outgoing and incoming telephone calls at the ego level. Here, for each ego and to avoid visual distraction and the influence of extreme values, we normalized the hourly fraction of telephone calls in the range (0, 1), where unity is the maximum fraction and zero is the minimum fraction (see Statistical Tools in the Methods section). The normalized results are displayed on a comparative heat map to illustrate the existence of various daily rhythms in the older adults participating in this study. Interestingly, for some egos, variations appeared between the outgoing and incoming peaks of telephone activity. For instance, individual D switched from a nocturnal peak in outgoing telephone activity to a morning peak in incoming telephone activity. Similarly, the outgoing telephone activity for egos S, T, and U peaked in the morning, whereas the incoming telephone activity peaked in the evening. These differences imply that the hours most propitious for social interaction depend on the social activity or social reactivity of the ego. Taken together, these observations emphasize the interesting characteristics of telephone activity, such as its potential to reflect the daily rhythms of social interactions in older people and to reveal possible discrepancies between social activity and reactivity.

### Evidence of Persistent Daily Rhythms in Both Outgoing and Incoming Telephone Calls in Older Adults

**Figure 4:**
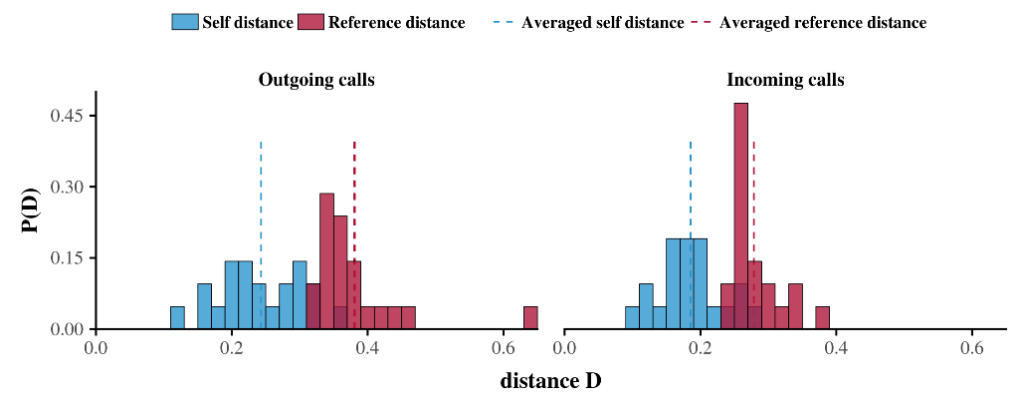
Average persistence histogram. Red bars represent the average reference distances, whereas blue bars represent the average self-distances for all egos in this study. Blue and red dashed lines represent the average reference distance and the average self-distance of the overall population, respectively. The results show that, on average, the self-distance of egos is less than their reference distance, which is evidence of circadian rhythms in telephone activity among older adults.

An approach based on the notion of persistence may be applied to analyze outgoing and incoming telephone calls. This approach was first introduced by Saramäki et al to analyze social signatures [44] and then used by Aledavood et al [32-34] to analyze circadian rhythms. At the individual level, the persistence of the daily rhythms calculated above was assessed by comparing the repeatability in time of each ego’s daily patterns (denoted *D*_self_) with a reference scale (denoted *D*_ref_) separately for the outgoing and incoming telephone calls (see Data Analysis in the Methods section). The results revealed the persistence of daily rhythms. In fact, for each ego, the average self-distance *D*_self_ was less than the reference distance *D*_ref_ for outgoing telephone calls. Similarly, the average *D*_self_ was less than *D*_ref_ for 20 of the 21 egos. Averaging the results for all egos provided 〈*D*_self_〉 = 0.24 (SD 0.06) and 〈*D*_ref_〉 = 0.38 (SD 0.07) for outgoing telephone calls, and 〈*D*_self_〉 = 0.19 (SD 0.03) and 〈*D*_ref_〉 = 0.28 (SD 0.04) for incoming telephone calls (Figure 4).

Additionally, we confirmed the persistence analysis by using a nonparametric statistical approach. To this end, for each ego, we compared the successive daily patterns of telephone call activity by applying Kolmogorov-Smirnov comparison tests to outgoing and incoming telephone calls separately, as previously performed [32]. The *P* values were strictly greater than .05 for 40 out of 42 cases concerning outgoing telephone calls, and after applying the Holm-Sidak method to correct for multiple comparisons [38], the adjusted *P* values were greater than .05 for all 42 cases. Similarly, for the incoming telephone calls, we obtain *P* values greater than .05 and adjusted *P* values greater than .05 for all 42 cases.

Taken together, these results indicate that, in general, the similarity in the egos’ daily rhythms cannot be rejected. Alternatively, a similar persistence analysis may be performed by using a classic Euclidean distance (*L*^2^) instead of the Jensen-Shannon divergence measure, as proposed previously [32]. Executing this alternative led to a similar conclusion for the rhythms of both the outgoing and incoming telephone calls (Table 2).

**Table 2:** Summary of averaged square root of Jensen-Shannon divergence and L2 metrics for the overall population for both reference distances and self-distances.

Variable		√JSD^a^ distance	*L*^2b^ distance
**Self-distance, mean (SD)**			
	Outgoing	0.24 (0.06)	0.14 (0.04)
	Incoming	0.19 (0.03)	0.14 (0.06)
**Reference distance, mean (SD)**			
	Outgoing	0.38 (0.07)	0.23 (0.07)
	Incoming	0.28 (0.04)	0.24 (0.11)

^a^JSD: Jensen-Shannon divergence.

^b^*L*^2^: classic Euclidean distance.

### Synchronization of Daily Rhythms for Outgoing and Incoming Telephone Calls at the Individual Level

**Figure 5:**
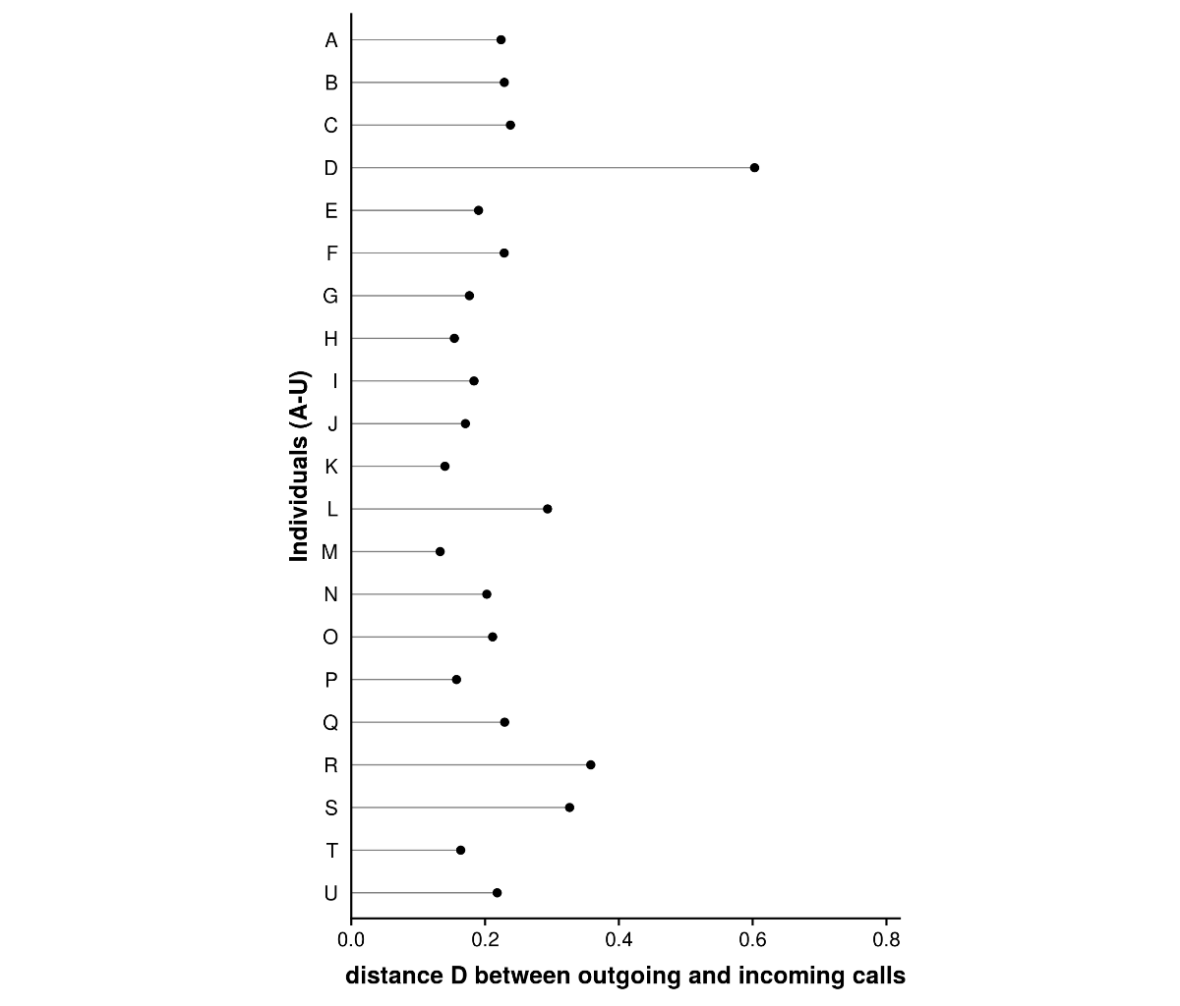
Distance D (ie, square root of Jensen-Shannon divergence) between the distributions of the outgoing and incoming telephone calls at the individual level and over the 12-month study period. The descriptive results show that dissimilarities exist between the daily rhythms for outgoing and incoming telephone calls of the various egos. In particular, although the daily rhythms of outgoing and incoming telephone call activity are highly dissimilar for ego D, the other individuals have dissimilarities that remain under a lower threshold.

Having thus far seen evidence that persistent daily rhythms exist in outgoing and incoming telephone calls and that these rhythms may correspond to circadian rhythm framing, we investigated any possible synchronization between these two daily rhythms. First, Figure 5 shows the square root of Jensen-Shannon divergence (see Statistical Tools in the Methods section) averaged over the 12-month study period for each ego to illustrate the synchronization between outgoing and incoming telephone calls at the individual level. The results revealed different dissimilarity values between the distributions of the outgoing and incoming telephone calls, which, except for one ego, tended to remain below a certain threshold.

We next applied a statistical approach based on the Kolmogorov-Smirnov comparison test under the null hypothesis “H_0_: the distributions of outgoing and incoming telephone calls follow the same law.” The results emphasized the similarity between these two distributions with a *P* value strictly greater than .05 for all 42 cases. These results can be explained by noting that, despite the variations between the daily rhythms of the outgoing and incoming telephone calls at the individual level, older people tended to distribute their outgoing and incoming communication time in the same way during the day. In short, a notable daily synchronization occurs in the way individuals place and receive telephone calls.

**Figure 6:**
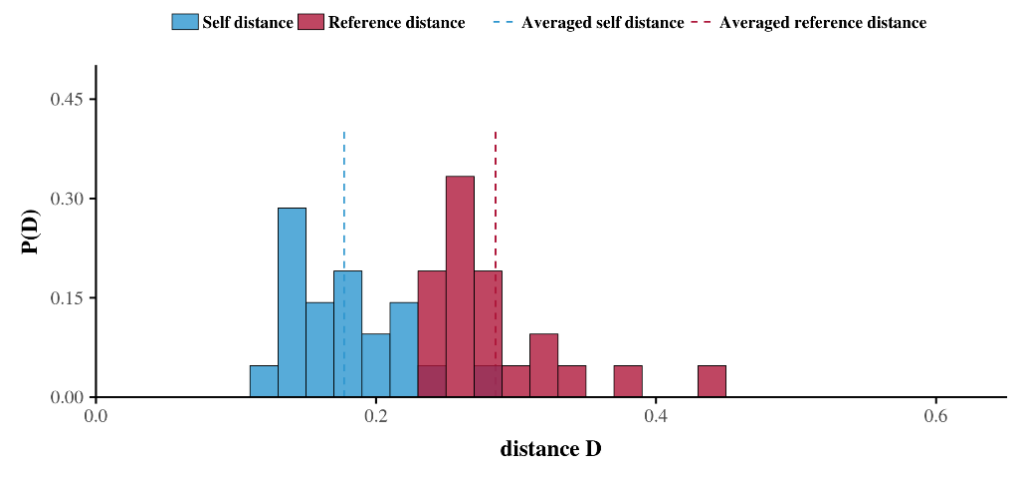
Histogram showing the average persistence for mixed outgoing-incoming telephone calls. Red bars represent the averaged reference distances, whereas blue bars represent the averaged self-distances for all the egos in the study. Blue and red dashed lines represent the averaged self-distance and the averaged reference distance of the overall population in the study, respectively. The results suggest that, on average, individuals have a self-distance that is less than their reference distance, which implies that, when mixing outgoing and incoming telephone calls without distinction, the daily rhythms of older adults persist over time.

One interesting consequence of this synchronization is that, at the individual level, when combining the outgoing and incoming telephone calls without distinguishing their direction, the resulting mixed daily pattern persists over time. In fact, to evaluate the persistence of these combined rhythms over time, we estimated the daily rhythms of the telephone call activity of each ego by merging together their outgoing and incoming telephone calls and removing the call direction. The results showed that, on average, 20 of the 21 individuals were persistent in their daily rhythms of telephone call activity. In addition, a statistical approach confirmed these results by comparing, for each ego and for each time period, the successive daily patterns of telephone call activity. Applying the Kolmogorov-Smirnov comparison test provided *P* values greater than .05 for all 42 cases (Figure 6). Taken together, these results show that, because of the synchronization, the daily rhythms of outgoing and incoming telephone activity can be merged without distinction. However, these results do not imply that the daily rhythms for outgoing and incoming telephone activity contain exactly the same information about how older people communicate with their social network during the day. Instead, older people have similar rhythms in their telephone activity and reactivity. Further, the alter specificity investigation designed by Aledavood et al [32] should provide useful insights.

### Alter Specificity Similarities in the Daily Rhythms of Outgoing and Incoming Telephone Calls

The results presented above show that persistent rhythms exist at the individual level for both outgoing and incoming telephone calls and that these rhythms are clearly synchronized with respect to the direction of the telephone call. However, these results do not provide alter specificity information about how older adults interact during the day with their social network. To clarify this situation, we used the method of Aledavood et al [32] to investigate whether alter specificity exists separately for outgoing and incoming telephone calls (see Data Analysis in the Methods section).

**Figure 7:**
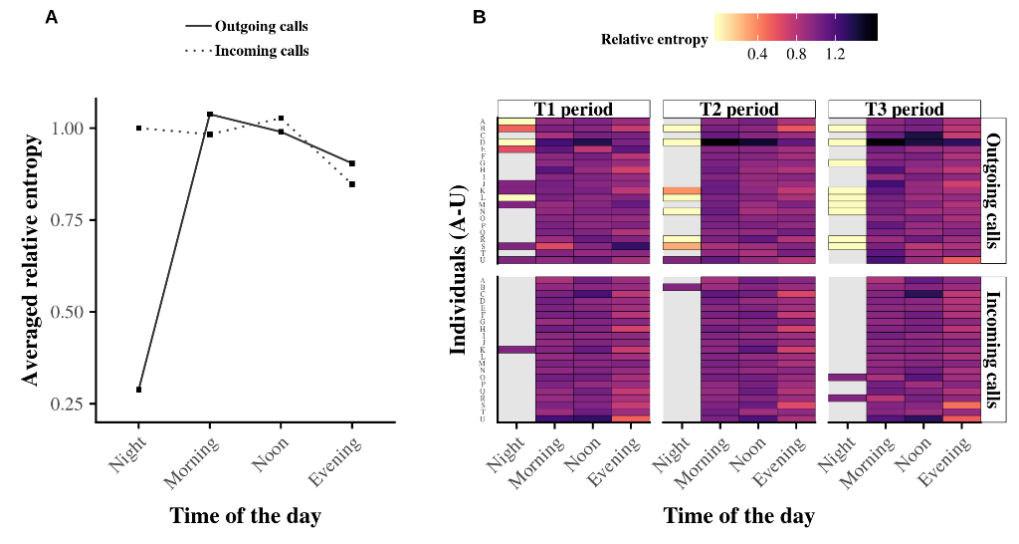
Relative entropy at the individual level. Panel A shows the averaged relative entropy for both outgoing (black curve) and incoming (dashed curve) telephone calls. Panel B summarizes the relative entropy of all 21 egos in a heat map. Light (dark) colors correspond to low (high) entropy, and gray indicates missing values. Although individuals have their own alter specificity during the daytime, the average relative entropy of the population is similar, except at nighttime when too much data are missing to obtain a significant result.

Overall, comparing the circadian rhythm of the outgoing telephone calls with that of the incoming telephone calls suggested that similarities exist for alter specificity. For both calling directions, the average relative entropy (see Data Analysis in the Methods section) of the population shown in Figure 7A indicated that egos tend to have low entropy in the evening and night but higher entropy in the morning and afternoon. However, this result did not hold for all egos, as shown in Figure 7B. Additionally, the incoming circadian rhythms at night nuance the relevance of these results because only four egos were active during this time interval. This low number of observations prevented us from concluding that the average entropy for this time period applies to the whole population.

**Figure 8:**
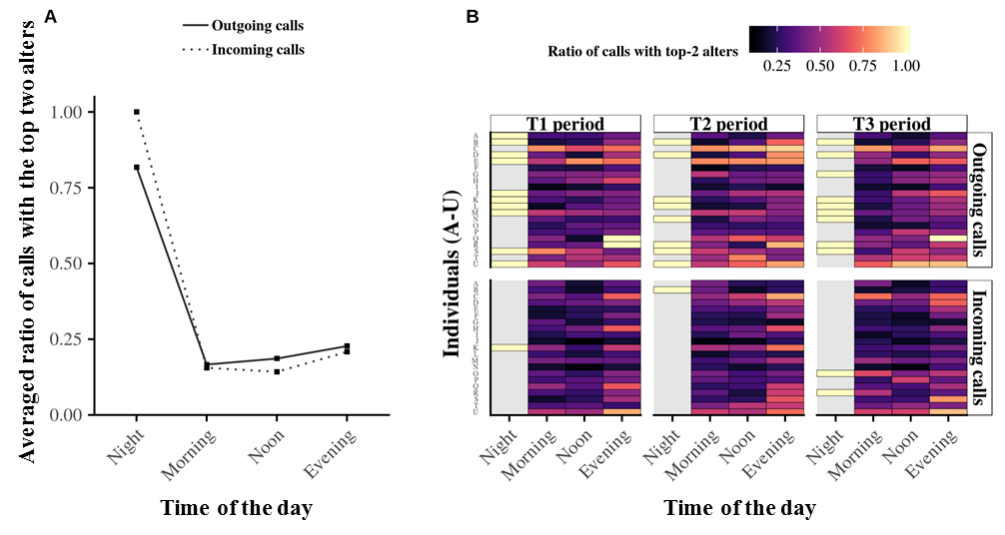
Fraction of telephone calls with the top two alters at the individual level. Panel A shows the average fraction of calls with the top two alters for both outgoing (black curve) and incoming (dotted curve) telephone calls. Panel B summarizes on a heat map the fraction of telephone calls with the top two alters for all 21 egos. A light (dark) color indicates a high (low) fraction, and gray indicates missing values. Although individuals have their own top two alter specificity during the day, the average relative entropy of the population seems similar, except at nighttime when too much data are missing to draw significant conclusions.

We next investigated whether particular rhythms exist at the individual level in communications involving the favorite telephone contacts of egos for both outgoing and incoming telephone calls. Figure 8 shows the specificity in telephoning to the top two contacts (ie, the two most-contacted alters), as performed by Aledavood et al [32]. The results suggest that similarities exist in how egos place telephone calls and respond to telephone calls with their two favorite alters during the day. Specifically, for outgoing and incoming telephone calls, the corresponding average pattern gives the maximum fraction of calls with the top two contacts in the evening and night, whereas it decreases in the morning and afternoon, as shown in Figure 8A. Additionally, this average fraction of calls also seems inversely proportional to the average relative entropy (Figure 7A). The heat map for the top two alters shown in Figure 8B confirmed that a large fraction of the telephone communications with the top two alters occurred at night. However, as discussed for Figure 7, because only four egos were contacted at night, we cannot extrapolate the resulting average entropy to the whole population.

**Figure 9:**
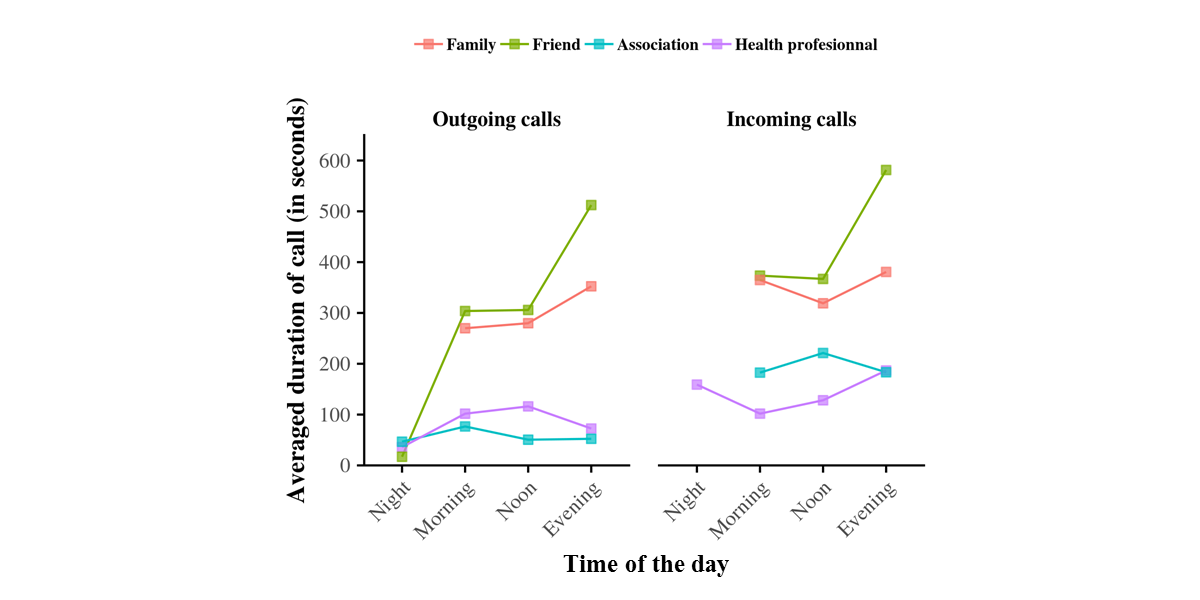
Average duration of telephone calls between egos and their social network during the day. Data show the average duration of telephone conversations between egos and their family (red), friends (green), health professionals (purple), and associations (blue).

Finally, we completed our investigation into alter specificity by considering the duration of telephone calls between egos and their social network during the day. Figure 9 shows the results. The following two results clearly stand out: (1) the duration of telephone calls tended to increase throughout the day until the night, when it dropped rapidly to a minimum and (2) on average, egos communicated longer with their close social network (family and friends) than with others (associations and health professionals). In fact, a threshold in call duration seemed to separate these two social circles.

## Discussion

### Principal Findings

Persistence and synchronization are important mechanisms that affect the daily rhythms of the social activities of older individuals. Unfortunately, they remain underinvestigated in the current literature on call detail record analysis. This work contributes to filling this gap by comparing estimates of circadian rhythms in call detail records of outgoing and incoming telephone calls of an older population. To this end, we focused on the following two issues: (1) the possibility of circadian rhythms existing in the outgoing and incoming telephone calling activities of seniors, and (2) the possible existence of synchronization between these two rhythms. This study used a data set of 12 successive months that combined call detail records and questionnaire data from 21 volunteers, all of whom were over 70 years of age. In particular, we extracted the following four specific telecommunication parameters from the call detail records: (1) recipient of the call (ie, alter), (2) time at which the call began, (3) duration of the call, and (4) direction of the call (outgoing or incoming).

The results indicated that differences exist between the daily rhythms of the salient features of outgoing and incoming telephone activities (eg, hourly peaks of calling) by older individuals (Figures 1-4) and that both of these rhythms are in accordance with circadian rhythm framing. Despite these differences, the daily rhythms of outgoing and incoming telephone calls generally follow the same distribution. In other words, the rhythms are synchronized. Interestingly, one consequence of this synchronization is that the daily rhythm persists upon combining outgoing and incoming telephone calls (Figure 6). Finally, based on a descriptive approach, we suggest that, behind this temporal synchronization, individuals also present similarities in the way that they place calls to and receive calls from specific alters during specific periods of the day (Figures 7-9).

Comparing the present findings on older adults with the existing literature on young individuals [32,33] reveals similarities in the way in which old and young individuals make telephone calls during the day. Both older adults and younger individuals exhibit consistent daily rhythms in their telephone activity in accordance with circadian rhythm framing. Furthermore, for both younger and older individuals, telephone calls made in the evening and night were more focused on specific well-known alters than those made during the rest of the day. However, particular differences also existed between these two populations. In particular, at the aggregate level, older adults exhibited general circadian rhythms of telephone activity with a bimodal distribution. Their telephone activity peaked in the morning and again in the evening, separated by a period of low activity in the afternoon. The circadian rhythm of the telephone activity of younger individuals, however, tends to follow a Gaussian distribution centered on the afternoon period [32,33].

This important difference between younger and older individuals may be explained by invoking the following four hypotheses:

(1) With increasing age, people tend to nap more in the afternoon, which could explain the period of low telephone activity in the older population studied herein [48].

(2) The work schedule for younger adults, as noted previously [32,33], may module their social activity and thus their telephone activity. Thus, specific periods of the day, such as after school, could be more propitious for telephone activity than other periods, such as the morning.

(3) In the older population, the chronotype shift that usually appears with age tends to make people more active in the morning, which could explain the first peak of telephone activity in the morning [17].

(4) Finally, cultural differences between the two samples, such as the country of origin of the participants (France in this study and the United Kingdom in previous studies [32,33]) may also impact the general circadian rhythms observed in the two studies, notably regarding cultural zeitgebers such as mealtimes and nap times.

Thus, taken together, the present results show that (1) older individuals may present persistent daily rhythms in telephone activity for both outgoing and incoming telephone calls in accordance with circadian rhythm framing and (2) the daily rhythm of their outgoing and incoming calls may be synchronized. These circadian rhythms contain both similarities and differences when compared with those of other populations, such as younger adults. In the field of health monitoring, these results suggest that the telephone may serve as a digital sensor of the temporal social activity of older individuals. In clinical practice, the telephone activity of older patients could be monitored to supervise their health in real-time based on their social activity and reactivity. Such supervision could consist of detecting particular health issues in progress, such as insomnia and other sleep disturbances that often occur with age [17], which could be signaled by nocturnal telephone activity. Additionally, such supervision could help to prevent risky social situations, such as isolation and depression, by detecting a decrease in the number and duration of outgoing and incoming telephone calls with individuals in the close social network of the patient. Both of these detections (ie, health issues and social risks) could be presented to health professionals in the form of triggers to help them better understand and anticipate health complications or worsening conditions of older patients. Notably, information stemming from monitoring telephone activity has the advantage of being collected in a passive and unobtrusive way. Such monitoring could be done in conjunction with traditional clinical questionnaires about the rhythms of social activity that are often used in clinical practice to monitor the daily behavior of patients [18]. Thus, this digital approach could potentially improve traditional health care systems by providing objective estimates of the social activity of patients to augment the subjective answers to questionnaires collected at more dispersed time intervals. However, before considering such an approach, the limitations described in the next section should be considered.

### Limitations

Because we provided a descriptive analysis based on a relatively rich but small sample of 21 older individuals, no straightforward generalization of the results should be made to the overall elderly population. In particular, given the potential sparsity of data and the small number of telephone calls made each day by the subjects in this study (Table 1), no foregone conclusions should be made regarding observations made under different conditions or on different data sets. To resolve this, future work should consider larger data sets and, more broadly, analyze different populations, such as healthy, disabled, or chronically ill individuals, or even vary the time period of observation. An evaluation of the reproducibility of the proposed approach would help to determine the robustness of the present results, which would in turn determine the feasibility of analyzing call detail records for health-monitoring purposes.

In addition, given the small sample size, the present quantitative data should be confronted with qualitative data. For health monitoring, such an approach could provide an interesting perspective to contextualize the present findings.

Finally, a technical limitation is present in the size of the time interval used in our data analysis for investigating the persistence and synchronization of circadian rhythms over time. Although no clear consensus exists in the literature about the size of the time interval when analyzing temporal telephone data, previous studies [41] have reported that aggregating data over long time intervals (eg, several months) limits the effect of short-term variations in call patterns, thereby enhancing the robustness of the persistence analysis. Consequently, by splitting our 12-month data set into three equal 4-month intervals as performed in previous work [32], the results for persistence and synchronization are valid only for a 4-month interval. For other intervals, further studies are required. Such investigations are welcome because call detail record analyses generally suffer from a lack of investigation into the reproducibility of the existing methodology to more general conditions, as mentioned previously [44].

### Conclusion

In this work, we estimated the circadian rhythms of the telephone activity of older adults. The results showed (1) temporal persistence and (2) synchronization between how older adults place and receive telephone calls over the day. At this point, more investigations are needed to determine the extent to which such rhythms may be harnessed for health-monitoring purposes. In particular, the results of this study showed that, despite high persistence and synchronization, differences exist between the daily rhythms of the outgoing and incoming telephone activities of older adults. These differences may relate, for example, to the daily peak hour in telephone activity.

### Perspectives

Determining the similarities and dissimilarities between the daily rhythms of outgoing and incoming telephone activities for health-monitoring purposes is of interest to health care professionals. At this point, however, the methodology used here, which is largely inspired by a previous report [32], does not permit us to determine the significance of the results. Along these lines, extracting relevant and important information from telephone activity for health monitoring is part of our immediate plan. We believe that such an approach could provide an opportunity for assessing the relevance of digital circadian rhythms to characterize the behavior of older patients and, more broadly, to investigate the role of new technologies in improving health care monitoring. Future studies should investigate, in particular, older adults with onset of neurodegenerative diseases, such as Alzheimer disease, for whom the persistence evinced here could be replaced by a transition to a pathologic perseveration that could be detected and characterized by repeated outgoing calls with a given circadian rhythm.
